# Genetic risk variants in the *CDKN2A/B, RTEL1* and *EGFR* genes are associated with somatic biomarkers in glioma

**DOI:** 10.1007/s11060-016-2066-4

**Published:** 2016-02-02

**Authors:** Soma Ghasimi, Carl Wibom, Anna M. Dahlin, Thomas Brännström, Irina Golovleva, Ulrika Andersson, Beatrice Melin

**Affiliations:** Department of Radiation Sciences, Oncology, Umeå University, Umeå, Sweden; Computational Life Science Cluster (CLiC), Umeå University, Umeå, Sweden; Department of Medical Biosciences, Pathology, Umeå University, Umeå, Sweden; Department of Medical Bioscience, Medical and Clinical Genetics, Umeå University, Umeå, Sweden

**Keywords:** CDKN2A/B, EGFR, RTEL1, SNP, FISH, ASCAT

## Abstract

During the last years, genome wide association studies have discovered common germline genetic variants associated with specific glioma subtypes. We aimed to study the association between these germline risk variants and tumor phenotypes, including copy number aberrations and protein expression. A total of 91 glioma patients were included. Thirteen well known genetic risk variants in *TERT, EGFR*, *CCDC26*, *CDKN2A*, *CDKN2B,PHLDB1*, *TP53*, and *RTEL1* were selected for investigation of possible correlations with the glioma somatic markers: *EGFR* amplification, 1p/19q codeletion and protein expression of p53, Ki-67, and mutated IDH1. The *CDKN2A/B* risk variant, rs4977756, and the *CDKN2B* risk variant, rs1412829 were inversely associated (*p* = 0.049 and *p* = 0.002, respectively) with absence of a mutated IDH1, i.e., the majority of patients homozygous for the risk allele showed no or low expression of mutated IDH1. The *RTEL1* risk variant, rs6010620 was associated (*p* = 0.013) with not having 1p/19q codeletion, i.e., the majority of patients homozygous for the risk allele did not show 1p/19q codeletion. In addition, the *EGFR* risk variant rs17172430 and the *CDKN2B* risk variant rs1412829, both showed a trend for association (*p* = 0.055 and *p* = 0.051, respectively) with increased *EGFR* copy number, i.e., the majority of patients homozygote for the risk alleles showed chromosomal gain or amplification of *EGFR*. Our findings indicate that *CDKN2A/B* risk genotypes are associated with primary glioblastoma without IDH mutation, and that there is an inverse association between *RTEL1* risk genotypes and 1p/19q codeletion, suggesting that these genetic variants have a molecular impact on the genesis of high graded brain tumors. Further experimental studies are needed to delineate the functional mechanism of the association between genotype and somatic genetic aberrations.

**Electronic supplementary material:**

The online version of this article (doi:10.1007/s11060-016-2066-4) contains supplementary material, which is available to authorized users.

## Introduction

Glioma includes several subtypes. Traditionally, they have been classified solely on histopathological features, though classification is currently changing towards accounting for molecular markers as well [1]. Previous studies have indicated that subtypes of glioma display separate molecular and genetic profiles resulting from their separate etiologic pathways. The somatic mutations and aberrations are sometimes correlated [2], such as the link between *IDH1* mutation and 1p/19q codeletion in low grade glioma [3–5]. Some of these markers, like *IDH1* mutation and *MGMT* methylation, have diagnostic value and are useful prognostic and predictive factors relating to patient survival and response to treatment [6–10]. 1p/19q codeletion is thought to be a distinguishing feature for oligodendroglioma and *TP53* mutations for astrocytoma, and even though they are not mutually exclusive, they are a clear support in the diagnostic classification [11]. *IDH1* mutations are known as an important diagnostic marker, especially for low graded tumors and secondary glioblastoma [12, 13]. In combination with loss of nuclear *ATRX* expression, *IDH1*, 1p/19q and *TERT* promoter mutations define the most frequent type of infiltrative astrocytoma [14, 15], while mutations in the *EGFR* gene (seen in 35 % of all cases of glioblastoma) are associated with primary glioblastoma [16]. In several of these genes that typically harbor somatic mutations in glioma, genome wide association studies (GWAS) have discovered common germline variants that are associated with risk of developing glioma, including variants in *EGFR*, *CDKN2A*, *TERT,* and *TP53* [17–22]. Furthermore, germline variants at 8q24.21 are known to be associated with oligodendroglial tumors and astrocytoma with mutated *IDH1* or *IDH2* [23]. Several single nucleotide polymorphisms (SNPs) have also been shown to associate with tumor grade. Variants in *CDKN2B* and *RTEL1* are strongly associated with high-grade glioma while variants in *CCDC26* and *PHLDB1* are associated with low-grade glioma [18, 24].

To investigate whether germline genetic risk variants are linked to specific molecular characteristics of the tumor, we selected 13 glioma risk variants established in the previous studies, mainly GWAS (Supplementary Table 1), and studied their correlation with the glioma somatic biomarkers: *EGFR* alteration, 1p/19q codeletion, *IDH1* mutation, p53 and Ki67 protein expression. We used immunohistochemistry (IHC) and fluorescence in situ hybridization (FISH) analyses to assess the biomarkers. In addition, FISH results were compared with the results from one of our previous studies, where somatic copy number data were calculated from SNP array [25] profiles, to explore if the different methods can detect similar genetic aberrations.

## Materials and methods

### Study population and tumor specimens

Paraffin-embedded glioma tissues were available from 91 patients for the present study, and the sample set and its characteristics are listed in Table 1. Histologically, 33 of the tumors were grade II-III glioma and 58 were glioblastoma (Table 1). The patients in the present study overlap with the ones included in a paper by Wibom et al. [25], where the ASCAT algorithm [26] was employed to calculate somatic genome-wide allele-specific copy number profiles (i.e., ASCAT profiles). The overlap is constituted by 59 patients that were included in both studies (Table 1; Supplementary Table 2). Informed consent was obtained from all individual participants included in the study. The ethical board approval was obtained for all experiments, in accordance with the Umeå University guidelines.

**Table 1:** Summary of patient characteristics

Total number of patients included in the study	91	Total number of patients included in the study, ASCAT	59
Median age (years)	58	Median age (years)	58
Age range (years)	15–80	Age range (years)	15–80
No. (%)		No. (%)	
Male	53 (58.2)	Male	35 (59.3)
Female	38 (41.8)	Female	24 (40.7)
Histological subtypes		Histological subtypes	
Pleomorphic xanthoastrocytoma grade II	1	Pleomorphic xanthoastrocytoma grade II	0
Astrocytoma grade II	2	Astrocytoma grade II	0
Astrocytoma grade III	12	Astrocytoma grade III	9
Oligodendroglioma grade II	9	Oligodendroglioma grade II	6
Oligodendroglioma grade III	7	Oligodendroglioma grade III	4
Oligoastrocytoma grade II	1	Oligoastrocytoma grade II	1
Ganglioglioma	1	Ganglioglioma	1
Glioblastoma	58	Glioblastoma	38

### Immunohistochemistry (IHC)

A neuropathologist identified histologically representative tumor regions that were stained by hematoxylin and eosin. Tissue sections were cut at 4 µm and the IHC was performed using the Ventana Benchmark system (Ventana Medical System, Tucson, AZ, USA). As a pre-treatment step, tissues were subjected to heat-induced epitope retrieval with the Cell Conditioning 2 solution (Ventana, Tucson, AZ, USA), 24 min for Ki-67 (30-9) (Ventana, Tucson, AZ, USA), 32 min for p53 (DO-7) (Ventana, Tucson, AZ, USA) and IDH1 (R132H) (Dianova, Hamburg, Germany). The antibody concentrations were 2 µg/ml for Ki-67, 184 µg/ml for p53, and 4 µg/ml for IDH1. Two independent observers evaluated the stained slides. Proliferation index was evaluated using Ki-67 antibody staining and calculated by determining the percentage of immunopositive nuclei. A total of 100-500 nuclei were counted. The tumors were divided into two groups, less aggressive (<15 %) and more aggressive ≥15 %). The consensus for p53 was scored in four different categories: no immunoreactivity (0 %), faint (≤50 %), moderate (50–75 %), and strong (≥75 %) immunoreactivity. IDH1 was scored in two categories: (0–10 %) for negative immunoreactivity, and (≥10 %) for positive immunoreactivity.

### Fluorescence in situ hybridization (FISH)

Tissue sections for 1p, 19q, and *EGFR* FISH staining were cut at 4 µm. The slides were deparaffinized, dehydrated, and placed in pretreatment solution (Vysis, Illinois, USA) followed by rinse in purified H_2_O and 2 × SSC. The slides were then treated for 45 min in 50 ml of solution (NaCl pH 2.0) containing 25 mg protease (Vysis, Illinois, USA), and rinsed in H_2_O and 2 × SSC. Locus-specific probes for *EGFR* (7p12), 1p36/1q13 and 19p13/19q13 were used as recommended by the manufacturer (Vysis, Illinois, USA). In short, probes were applied and a coverslip was placed over the target area, followed by sealing with rubber cement to prevent evaporation of the probe. Simultaneous denaturation of the probe and target was carried out on the THERMOBrite (Abbott Molecular, Illinois, USA) at 74 °C for 6 min. Hybridization was performed by placing the slides in a humidified chamber at 37 °C for overnight incubation. After hybridization, slides were treated in a post-hybridization wash of 2 × SSC solution containing 0.3 % NP40 at 73 °C and nuclei were counterstained by DAPI (Sigma-Aldrich, USA) nuclear counterstain. Antifade (CitiFluor, London, UK) was applied and the sections were viewed using a Zeiss Axio Imager Z1fluorescent microscope with a dual green/orange filter (Vysis, Illinois, USA). Three observers evaluated the slides and the evaluation was based on 100 intact non-overlapping nuclei that were counted for both the green and orange signals. The ratio of *EGFR* was calculated using the criteria developed in previous studies [27–29]. A ratio between the locus specific probe (*EGFR*) and the control probe *CEP7* (*EGFR*/*CEP7*) was calculated where ratios equal to 1 was considered as normal, while more than 10 % cells with a ratio between 1 and 2 was considered as chromosomal gain and more than 10 % cells with a ratio greater than 2 was considered as amplification. The ratio between the locus specific probe and control probe for both 1p (1p36/1q25) and 19q (19q13/19p13) was calculated using the criteria used in the clinical routine practice [30], 1p36/1q25 ratios < 0.88 and 19q13/19p13 ratios < 0.74 in more than 12 % of the cells were considered as deleted.

### SNP array

Data was taken from our previous study [25] where DNA was extracted from glioma tissue using QIAmp Mini Kit (QIAGEN GmbH, Hilden, Germany) and genotyped using Illumina HumanOmni1-Quad BeadChips. The ASCAT algorithm [26] (version 2.0) was used to calculate somatic whole-genome allele-specific copy number profiles (ASCAT-profiles), as well as estimates of tumor cell content and tumor cell ploidy. For comparison between FISH and ASCAT, we extracted the median total copy number from the ASCAT profiles for the genomic regions corresponding to the FISH probes. These copy number data were subsequently used to mimic the sample classification based on FISH data, by calculating the same ratios and using the same cutoff values that had been used for classification by FISH. More details about the SNPs can be found in supplementary Table 1 and samples included in analyses with both FISH and ASCAT are shown in Table 1.

### Statistical analyses

The associations between the biomarkers and genetic risk variants as well as comparisons of different methods were evaluated using the *χ*^2^ test or the Fisher’s exact test. The significance level was set at *p* < 0.05. Six genetic variants (rs2252586, rs17172430, rs11979158, rs4295627, rs55705857, and rs78378222) were not genotyped by the SNP array. Therefore, these variants were imputed using the software IMPUTE2 with data from the 1000 Genomes Project as the reference population. One SNP, rs55705857 was excluded from further analysis since it could not be imputed with high certainty (imputation score < 0.80) (Supplementary Table 1).

## Results

Eighty glioma patients were successfully analyzed for *EGFR* copy number variation and 1p/19q codeletion, however two samples were excluded since the ratio was below 1 and there were too few patients to make a separate group for these two samples. *EGFR* amplification was observed in 24 of 78 (30.8 %) glioma tumors and in 18 of 47 (38.3 %) glioblastoma tumors. 1p/19q codeletion was observed in 14 of 78 (17.9 %) glioma tumors and 8 of 50 (16.0 %) glioblastoma tumors. Due to lack of patient material and failed analyses different numbers of glioblastoma tumors are analyzed for *EGFR* amplification and 1p/19q codeletion (Table 2).

**Table 2:** Protein expression by means of IHC staining and copy number variation by means of FISH analysis for the glioma biomarkers

Glioma biomarkers	Number (%)
Ki67^a^	
<15 %	46/91 (50.5)
>15 %	45/91 (49.5)
IDH1 (R132H), total^a^	
Negative	75/90 (83.3)
Positive	15/90 (16.7)
IDH1 (R132H), glioblastoma^a^	
Negative	53/57 (93.0)
Positive	4/57 (7.0)
p53, total^a^	
Negative	4/89 (4.5)
Faint + moderate	58/89 (65.2)
Strong	27/89 (30.3)
p53, glioblastoma^a^	
Negative	1/56 (1.8)
Faint + moderate	38/56 (67.9)
Strong	17/56 (30.3)
*EGFR,* total^b^	
Normal	15/78 (19.2)
Chromosomal gain	39/78 (50.0)
Amplification	24/78 (30.8)
*EGFR*, glioblastoma^b^	
Normal	5/47 (10.6)
Chromosomal gain	24/47 (51.1)
Amplification	18/47 (38.3)
1p/19q, total^b^	
Codeletion	14/78 (17.9)
No codeletion	64/78 (82.1)
*1p*/19q, glioblastoma^b^	
Codeletion	8/50 (16.0)
No codeletion	42/50 (84.0)

Ki67 proliferation index was scored for percentage of positive nuclei in a cell population and dived into less aggressive (<15 %) and more aggressive (>15 %) groups. IDH1 protein expression was scored as (0–10 %) for negative, and (>10 %) for positive immunoreactivity and p53 protein expression was scored as (0 %) for negative, (25–50 %) for faint, (50–75 %) for moderate (since there were too few cases in this group, faint and moderate expression was merged as one group for statistical analysis), and (>70 %) for strong immunoreactivity. Due to lack of patient material and failed analyses different numbers of samples are analyzed for the different biomarkers

^a^Immunohistochemistry (IHC) staining

^b^Fluoroscence in situ hybridization (FISH) analysis

The blood samples corresponding to the tumor samples were analyzed with the SNP array. Four genetic risk variants showed association with the investigated glioma biomarkers (Table 3). The *CDKN2A/B* risk variant (rs4977756) and the *CDKN2B* risk variant (rs1412829) were both inversely associated with expression of mutated IDH1 (*p* = 0.049 and *p* = 0.002, respectively) since for both these variants, the majority of patients homozygous for the risk allele (*G*) showed no or low (0–10 % immunoreactivity) expression of mutated IDH1. The *CDKN2B* risk variant, rs1412829 and the *CDKN2A/B* risk variant, rs4977756 are both located on chromosome 9p21 within the same gene cluster as the non-coding RNA CDKN2B-AS1 (also known as ANRIL), and these risk variants are largely dependent of each other in terms of linkage disequilibrium (LD) since they are both located within the same haplotype block (r^2^ = 0.741; D′ = 0.888). The *RTEL1* risk variant (rs6010620) was inversely associated with 1p/19q codeletion (*p* = 0.013) since the majority of patients homozygous for the risk allele (*G)* showed no 1p/19q codeletion. In addition, we observed a trend of higher frequency of *EGFR* amplified tumors in patients homozygous for the *EGFR* risk variant (rs17172430) and the *CDKN2B* risk variant (rs1412829). This finding was however not statistically significant. None of the other evaluated risk variants showed any significant associations with the investigated glioma biomarkers.

**Table 3:** Association between genetic risk variants and molecular alteration

Mutated IDH1, IHC	Negative (%)	Positive (%)	*p* value
*CDKN2A/2B_rs4977756*			
AA	14 (66.7)	7 (33.3)	0.049
AG	34 (91.9)	3 (8.1)	
GG	20 (83.3)	4 (16.7)	
AG + GG	54 (88.5)	7 (11.5)	0.022

Mutated IDH1, IHC	Negative (%)	Positive (%)	*p* value
*CDKN2B_rs1412829*			
AA	10 (55.6)	8 (44.4)	0.002
AG	38 (92.7)	3 (7.3)	
GG	20 (87.0)	3 (13.0)	
AG + GG	58 (90.6)	6 (9.4)	0.0005

*1p/19q* loss, FISH	No codeletion (%)	Codeletion (%)	*p* value
*RTEL1_6010620*			
GG	38 (84.4)	7 (15.6)	0.013
AG	17 (85.0)	3 (15.0)	
AA	1 (25.0)	3 (75.0)	
AG + AA	18 (75.0)	6 (25.0)	0.339

*EGFR*, FISH	Normal (%)	Chromosomal gain (%)	Amplification (%)	*p* value
*CDKN2B_rs1412829*				
*AA*	1 (7.1)	11 (78.6)	2 (14.3)	0.051
*AG*	9 (25.0)	17 (47.2)	10 (27.8)	
*GG*	2 (9.1)	9 (40.9)	11 (50.0)	
*AG* + *GG*	11 (19.0)	26 (44.8)	21 (36.2)	0.076

*EGFR*, FISH	Normal (%)	Chromosomal gain (%)	Amplification (%)	*p* value
*EGFR_rs17172430*				
GG	11 (21.1)	21 (40.4)	20 (38.5)	0.055
AG	0 (0.0)	11 (78.6)	3 (21.4)	
AA	0 (0.0)	2 (100.0)	0 (0.0)	
AG + AA	0 (0.0)	13 (81.2)	3 (8.8)	0.017

Samples were classified as positive or negative for expression of mutated IDH1 based on the percentage of positive nuclei; ≤10 % for negative and >10 % for positive. *1p36*/*1q25* ratios <0.88 and *19q13*/*19p13* ratios <0.74 in more than 12 % of the cells were considered as codeleted. *EGFR* copy number aberrations were classified based on the *EGFR*/*CEP 7* ratio; ratio = 1 was classified as normal, ratio between 1 and 2 in >10 % of the cells was classified as gain, ratio >2 in >10 % of the cells was classified as amplified. The total number of samples listed for each association may differ, due to missing genotype data

*IHC* Immunohistochemistry, *FISH* fluorescence in situ hybridization

To compare the copy number profiles achieved by applying ASCAT to SNP array data with results from the FISH analysis, we focused on 1p/19q codeletion and *EGFR* amplification, because these features have clinical implications. For 1p/19q codeletion, there were 55 patients with data from both methods available, and 59 patients with data from both methods were available for *EGFR* amplification. The comparison yielded entirely disparate results with regards to 1p/19q codeletion, where FISH detected 14 samples displaying this aberration whereas none was detected based on SNP array data (Supplementary Table 3). The similarity in results from the two techniques was greater with regards to *EGFR* amplification. Using FISH, we detected 24 samples with *EGFR* amplification, of these 23 had ASCAT profiles available and 17 of them displayed *EGFR* amplification also by the SNP array approach (Table 4). In addition, 3 samples displayed chromosomal gain in *EGFR* as analyzed by FISH, of these 2 had ASCAT profiles available but none of them displayed chromosomal gain in *EGFR* also by the SNP array approach (Table 4).

**Table 4:** Patients displaying chromosomal gain and amplification in *EGFR* as observed by FISH analysis and results from corresponding analyses on ASCAT profiles

Patients	Diagnose	Number of cells (%) with chromosomal gain in EGFR (FISH analysis)	Number of cells (%) amplified in EGFR (FISH analysis)	Patients available in ASCAT dataset	No	Genetic abberation in EGFR (ASCAT algorithm)	Amplification
				Yes/No		Chromosomal gain	
1	Glioblastoma		90	Yes			X
2	Glioblastoma		80	Yes			X
3	Glioblastoma		100	Yes			X
4	Glioblastoma		100	Yes			X
5	Oligodendroglioma grade III		100	Yes			X
6	Glioblastoma		85	Yes			X
7	Glioblastoma		100	Yes			X
8	Astrocytoma grade III		100	No			
9	Oligodendroglioma grade III		95	No			
10	Glioblastoma		85	Yes			X
11	Oligodendroglioma grade II		65	Yes			X
12	Glioblastoma		100	Yes			X
13	Glioblastoma		91	Yes	X		
14	Astrocytoma grade III		100	Yes		X	
15	Oligodendroglioma grade III	55		No			
16	Glioblastoma		97	No			
18	Astrocytoma grade III	86		Yes	X		
19	Glioblastoma		35	Yes	X		
20	Glioblastoma	30		Yes	X		
21	Glioblastoma		69	Yes			X
22	Glioblastoma		40	Yes			X
23	Glioblastoma		100	Yes		X	
24	Glioblastoma		100	Yes			X
25	Glioblastoma		90	Yes			X
26	Glioblastoma		100	Yes			X
27	Glioblastoma		100	Yes			X
28	Glioblastoma		100	Yes			X

**Fig. 1:**
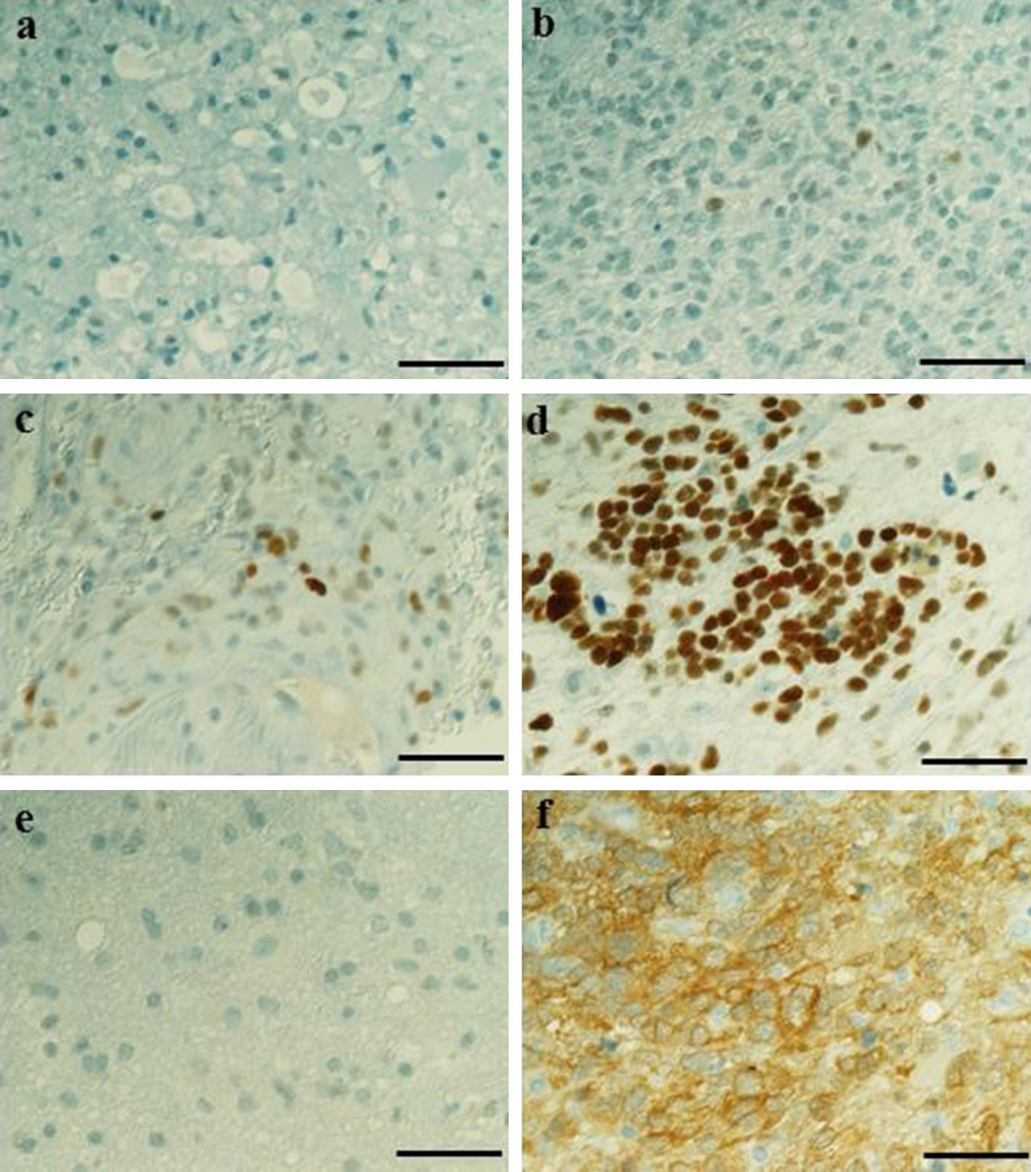
Immunohistochemical staining for p53 and mutated IDH1. Expression of p53 was scored in four different categories: **a** negative, **b** faint expression, **c** moderate expression, **d** strong expression. Expression of mutated IDH1 was scored for either **e** negative, or **f** positive

Based on proliferation index, 46 of 91 glioma tumors were considered less aggressive and 45 of 91 were more aggressive. Expression of mutated IDH1 was found in 15 of 90 glioma tumors, whereas 4 of 57 cases in the glioblastoma subgroup were positive for mutated IDH1. Almost all glioma patients, 85 of 89, showed p53 expression. In the glioblastoma subgroup, 38 of 56 showed faint to moderate protein expression while 17 patients demonstrated strong p53 protein expression (Fig. 1). Due to lack of patient material and failed analyses different numbers of samples are analyzed for the different biomarkers.

## Discussion

There are specific molecular markers in glioma characterization used to define the histological subtypes and grades of malignancy, as well as markers of diagnostic and prognostic value, and markers that may be used to predict response to treatment. Exploring an association between germline genetic variation and molecular alterations could be a key for definition of unique molecular based subtypes of glioma.

Previous studies have observed that some genetic variants are associated with tumor grade, like risk variants in the *CDKN2B*, *RTEL1*, and *TERT* regions [18, 31], which show association with high grade glioma, while risk variants in the *CCDC26* and *PHLDB1* regions are associated with low grade glioma involving *IDH* mutation, and 1p/19q codeletion [17, 31]. Although, association with tumor grade was not analyzed in our study due to the small number of low grade glioma, we found two risk variants in the *CDKN2A* and *CDKN2B* regions associated with mutated IDH1 (Table 3). The risk variant near *CDKN2B* (rs1412829) is the same risk variant associated with tumor grade in the study by Wrensch et al. [18]. We found expression of mutated IDH1 in few glioblastoma cases, which is in concordance with previous studies [4]. These findings might have clinical implications as a potential predictive marker, since recently updated data from the RTOG 9402 trial showed that *IDH* mutations predict the benefit of adjuvant chemotherapy in grade III glioma [32]. Other studies have shown that oligodendroglial tumors and glioma with mutated *IDH1* are strongly associated with the chromosome 8q24.21 risk variant (rs55705857) [23]. Conversely, and probably due to low statistical power in our study, we do not see any strong association between *IDH1* mutations and the chromosome 8q24.21 risk variant. One risk variant in *RTEL1* (rs6010620) that previously has shown association with 1p/19q codeletion [31], was significantly associated with 1p/19q codeletion also in our study. It has earlier been shown that genetic variants within or near the *RTEL1* (20q13) regions are strongly associated with glioblastoma [33]. *RTEL1* has been hypothesized to be involved in the resolution of D loops that occur during homologous recombination, and is together with *TERT* supposed to play a role in regulating telomere length [34, 35]. We found an inverse association between 1p/19q codeletion and the risk variant in *RTEL1* (rs6010620) but not the risk variant in *TERT* (rs2736100). Although the number of patients homozygous for the non-risk genotype in this comparison was only 4, our results are in line with previous studies, and suggest that germline glioma risk variants might be involved in the development and progression of high grade glioma. Nevertheless, since the majority of the genetic variants analyzed in this study are located in introns or intergenic regions, and do not result in amino acid changes in transcribed proteins, the mechanism of action behind these associations need to be further elucidated.

We have previously shown that two risk variants (rs17172430 and rs11979158) in *EGFR* are associated with homozygous deletion at the *CDKN2A/B* locus, and that one of the risk variants (rs17172430) in *EGFR* also shows association with allele specific loss of heterozygosity at the *EGFR* locus [25]. In this study, both the *EGFR* risk variant (rs17172430) and the *CDKN2B* risk variant (rs1412829) showed a trend for an association with chromosomal gain and amplification in *EGFR*. Similar trends were observed in the same sample set based on ASCAT copy number profiles, but they did not validate when tested on a TCGA data set in our previous study [25]. The association with chromosomal gain might indicate that these genotypes are associated with increased genetic instability where the tumor is more prone to have genetic aberrations with loss of one allele and copy number increase of the remaining allele. The genetic variants in *EGFR* that have been associated with glioma risk are not closely linked in the genome, and therefore these genotypes could give disparate result. In this study, the sample number is relatively small and thus suffering from limited statistical power to detect associations, particularly affecting low-frequency variants and variants with small effect size. The genotype-phenotype associations are not significant following adjustment to the family-wise error rate (Bonferroni correction). However, this procedure to adjust for multiple testing might be too stringent given that some investigated variables in this study are not independent. Larger glioma studies with dense tagging of the *EGFR* gene are required to elucidate the number of true associated genetic variants.

In addition, we have compared the present study with a previous study, where ASCAT profiles were calculated on a set of samples that overlapped with the samples included in this study. We observed that the different methodologies identifies dissimilar types of genetic aberrations. The SNP array approach cover the whole genome but might be considered less sensitive than FISH to detect aberrations in tumor subclones. For 1p/19q codeletion, the aberrations that the FISH analysis detected was not identified by the ASCAT analysis (data not shown), while for *EGFR*, results from the two methods showed a better correlation (Table 4). Both methods compared in this study have advantages and disadvantages. Establishment of a good threshold level for positive results is important for avoiding over interpretation of small cell populations when using FISH analysis and SNP array. However, the threshold for 1p/19q codeletion is well established in the clinic [30] and the threshold of *EGFR* amplification is well studied [27–29]. The FISH analysis technique uses fluorescently labeled DNA probes to detect chromosomal abnormalities. Applying ASCAT to SNP array data allow us to estimate both tumor cell content and tumor cell ploidy, which cannot be detected by FISH analysis. A uniparental disomy, when cancer cells have lost one chromosome in the presence of duplication of the other chromosomal allele, cannot be detected by FISH analysis, while this can be detected by ASCAT. FISH analysis with locus-specific probe does not allow testing for multiple chromosomal loci which can be detected by SNP arrays. On the other hand, the ASCAT algorithm assumes that the tumor sample is from the same clone and will ignore the heterogeneity of the tumor, which is a well-known aspect of glioma and this could be an explanation why ASCAT fails to detect 1p/19q codeletion.

In conclusion, even though the results need to be taken with caution since this study represents a small sample size, we found inverse associations between genetic risk variants in *CDKN2A/2B*, *RTEL1IDH1* mutation and 1p/19q codeletion, in line with previous studies. Whereas the results revealing that risk variants in *EGFR* and *CDKN2B* both showed a trend for association with *EGFR* copy number variation are new findings. The idea that the genetic variants could be used as a complementary diagnostic approach for tumors difficult to assess for conclusive biopsies is an interesting diagnostic concept in glioma, where there seem to be a limited number of genetic predisposing loci and robust biomarkers that might be added to diagnostics.

## Electronic supplementary material

Below is the link to the electronic supplementary material.

Supplementary material 1 (DOCX 19 kb)

Supplementary material 2 (XLSX 16 kb)

Supplementary material 3 (DOCX 21 kb)
