# Supplementary material for: Genetic risk variants in the CDKN2A/B, RTEL1 and EGFR genes are associated with somatic biomarkers in glioma
Source: J Neurooncol. 2016 Feb 2;127:483–92. doi: 10.1007/s11060-016-2066-4 (PMC4835517; doi:10.1007/s11060-016-2066-4)
Supplement: Supplementary file 1 — Supplementary material 1 (DOCX 19 kb) [file 11060_2016_2066_MOESM1_ESM.docx]

**Supplementary Table 1** Risk variants from previous studies that were selected for investigation in this study.

| **Risk variant** | **Chr** | **Gene** | **Major**  **allele** | **Risk**  **allele** | **Imputation**  **Score** | **Reference** |
| --- | --- | --- | --- | --- | --- | --- |
| rs2736100 | 5 | *TERT* | A | C |  | Shete S et al 2009 |
| ^1^rs2252586 | 7 | *EGFR* | G | A | 0.939 | Sanson M et al 2011 |
| rs6969537 | 7 | *EGFR* | G | G |  | Schwartzbaum JA et al 2010 |
| ^1^rs17172430 | 7 | *EGFR* | G | G | 0.944 | Andersson U et al 2010 |
| ^1^rs11979158 | 7 | *EGFR* | A | A | 0.997 | Sanson M et al 2011 |
| rs4947979 | 7 | *EGFR* | A | A |  | Andersson U et al 2010 |
| ^1^rs4295627 | 8 | *CCDC26* | A | G | 0.999 | Shete S et al 2009 |
| rs1412829 | 9 | *CDKN2B* | A | G |  | Wrensch M et al 2009 |
| rs4977756 | 9 | *CDKN2A/B* | A | G |  | Shete S et al 2009 |
| rs498872 | 11 | *PHLDB1* | G | A |  | Shete S et al 2009 |
| ^1^rs55705857* | 11 | *PHLDB1* | A | G | 0.535 | Jenkins RB et al 2012 |
| ^1^rs78378222 | 17 | *TP53* | A | C | 0.801 | Stacey SN et al 2011 |
| rs6010620 | 20 | *RTEL1* | G | G |  | Shete S et al 2009, Wrensch M et al 2009 |

^1^Risk variants not available on the SNP array were imputed. *****SNPs with imputation score <0.80 were excluded from the statistical analyses.

**Journal name:**

Journal of Neuro-oncology

**Title:**

Genetic risk variants in the *CDKN2A/B, RTEL1* and *EGFR* genes are associated with somatic biomarkers in glioma

Soma Ghasimi^1^ • Carl Wibom^1,2^ • Anna M. Dahlin^1,2^ • Thomas Brännström^3^ • Irina Golovleva^4^ • Ulrika Andersson^1^ • Beatrice Melin^1^

^1^Department of Radiation Sciences, Oncology, Umea University, Umea, Sweden

^2^Computational Life Science Cluster (CLiC), Umea University, Umea, Sweden

^3^Department of Medical Biosciences, Pathology, Umea University, Umea, Sweden

^4^Department of Medical Bioscience, Medical and Clinical Genetics, Umea University, Umea, Sweden

Corresponding author: [Ulrika](mailto:Ulrika) Andersson, E-mail: [ulrika.l.andersson@umu.se](mailto:ulrika.l.andersson@umu.se), Phone: +46 90 785 28 65.
