# Supplementary material for: Genetic risk variants in the CDKN2A/B, RTEL1 and EGFR genes are associated with somatic biomarkers in glioma
Source: J Neurooncol. 2016 Feb 2;127:483–92. doi: 10.1007/s11060-016-2066-4 (PMC4835517; doi:10.1007/s11060-016-2066-4)
Supplement: Supplementary file 3 — Supplementary material 3 (DOCX 21 kb) [file 11060_2016_2066_MOESM3_ESM.docx]

**Supplementary Table** **3** Patients displaying codeletion in 1p/19q region as observed by FISH analysis and results from corresponding analyses on ASCAT profiles.

**Journal name:**

Journal of Neuro-oncology

**Title:**

Genetic risk variants in the *CDKN2A/B, RTEL1* and *EGFR* genes are associated with somatic biomarkers in glioma

Soma Ghasimi^1^ • Carl Wibom^1,2^ • Anna M. Dahlin^1,2^ • Thomas Brännström^3^ • Irina Golovleva^4^ • Ulrika Andersson^1^ • Beatrice Melin^1^

^1^Department of Radiation Sciences, Oncology, Umea University, Umea, Sweden

^2^Computational Life Science Cluster (CLiC), Umea University, Umea, Sweden

^3^Department of Medical Biosciences, Pathology, Umea University, Umea, Sweden

^4^Department of Medical Bioscience, Medical and Clinical Genetics, Umea University, Umea, Sweden

Corresponding author: [Ulrika](mailto:Ulrika) Andersson, E-mail: [ulrika.l.andersson@umu.se](mailto:ulrika.l.andersson@umu.se), Phone: +46 90 785 28 65.
